# Do Mental Health Outpatient Services Meet Users’ Needs? Trial to Identify Factors Associated with Higher Needs for Care

**DOI:** 10.1007/s10597-015-9923-z

**Published:** 2015-09-21

**Authors:** Ewelina Dobrzynska, Joanna Rymaszewska, Przemyslaw Biecek, Andrzej Kiejna

**Affiliations:** Department of Psychiatry, Wrocław Medical University, ul. Pasteura 10, 50-367 Wrocław, Poland; Cygnet Hospital Kewstoke, Beach Road, Weston-super-Mare, BS22 7FD UK

**Keywords:** Needs, Mental disorders, Outpatient mental health services, Social functioning

## Abstract

The study was conducted to investigate the extent to which services meet patients’ needs and identify the factors associated with higher needs. 174 outpatients were assessed using CANSAS, BPRS and GSDS. The total number of unmet needs in persons with psychotic, eating, personality and affective disorders was higher than in patients with anxiety disorders. Being single, positive symptoms, depression/anxiety, hospitalizations and high social disability accounted for 50 % of the variance in level of unmet need. Persons with eating and personality disorders reported similar level of unmet needs to those with psychotic and affective disorders. The best correlates of unmet needs were depression/anxiety and social disability.

## Introduction

Mental disorders belong to a group of diseases with the most severe impact on social functioning. The increasing number of patients with disability poses a serious social, medical and economic problem. The failure of psychiatric deinstitutionalization reforms and a tendency to limit the costs of care have initiated the idea of creating mental health services on the basis of users’ needs. A current definition of need published by the National Health Service indicates that need is “the capacity to benefit from health care services”. Needs can be assessed from different perspectives, including staff, patient or carer, and have been differentiated into unmet needs (posing a current serious problem, whether or not help is given) and met needs (meaning no or moderate problem because of help given) (Phelan et al. 1995).

Patients’ satisfaction with services is related to the extent to which general health care needs and condition-specific needs are met. Leese et al. (1998) have found that satisfaction with mental health services has a strong correlation with unmet needs as well as with the duration of contact with services. Moreover, evaluating to what extent patients are satisfied with health services is proved to be clinically relevant. Berghofer et al. (2001) have argued that there is a relationship between satisfaction with services and quality of life, social functioning and one year prognosis. Satisfied patients are more likely to comply with treatment (Guldvog 1999), take an active role in their own care (Donabedian 1988) and continue using medical care services (Marquis et al. 1983). Therefore, they generally ought to have a better outcome.

Most of the studies on needs with regard to mental health patients refer to persons with schizophrenia and schizophrenia spectrum disorders only. There is a clear shortage of studies including persons with different mental disorders, especially those with personality and eating disorders. Assessing and predicting the needs of patients with different diagnoses could enable more adequate care planning, therapy and rehabilitation, which would consequently lead to better treatment outcome and better social functioning of service users.

## Aim

This study was conducted to investigate the extent to which Polish mental health outpatient services meet patients’ needs and to identify the variables (socio-demographic and clinical, including social functioning) associated with higher needs in persons with mental disorders.

## Subjects and Methods

### Participants

Data were collected from patients attending four mental health outpatient clinics based in Lower Silesia province, Poland. The sample consisted of patients aged 18–64 with minimum 6 month duration contact with psychiatric services and diagnosis F2-6 according to ICD-10 (F2 psychotic, F3 affective, F4 anxiety, F5 eating and F6 personality disorders). Excluding criteria were organic mental disorders, mental and behavioural disorders due to psychoactive substance use as well as serious and chronic somatic disorders. Additional excluding criteria included receiving Disability Living Allowance or pension as the main objective of the study was to evaluate the needs of psychiatric outpatients potentially work active, not already disabled.

Socio-demographic factors such as age, gender, employment status, marital status as well as clinical variables including diagnosis according to ICD-10, length of contact with psychiatric services, number of psychiatric hospitalizations, current pharmaco- and psychotherapy were all obtained from medical records.

Characteristics of the sample are shown in Table 1. In total, 174 service users were recruited and their mean age was 35 years (SD = 11.2). The majority of participants were women (n = 125, 72 %). Regarding marital status and employment, the majority of participants were in formal relationships (n = 70, 40 %) and either working full time or studying (n = 111, 64 %). The highest number of service users were those with anxiety disorders (F4 according to ICD-10) (n = 57, 33 %), and the least common diagnosis was eating disorder (F5). In the group of patients with psychotic disorders (F2), 80 % had been diagnosed with schizophrenia. Among all recruited patients, 41 service users (24 %) suffered from additional somatic disorder. Mean length of contact with psychiatric mental health services was 5 years (SD 6.2). The total number of patients who had been hospitalized previously was 40 (23 %) and the majority of them had been diagnosed with a disorder from group F2.

**Table 1:** Socio-demographic and clinical characteristics of the sample

1. Gender	
Male	49 (38 %)
Female	125 (72 %)
2. Marital status	
Single	61 (35 %)
In relationship	93 (53 %)
Separated	15 (9 %)
Widow/er	5 (3 %)
3. Employment status	
Full time work/student	111 (64 %)
Part time work	13 (7 %)
Unemployed	50 (29 %)
4. Diagnosis	
F2 psychotic disorders	29 (17 %)
F3 affective disorders	41 (23 %)
F4 anxiety disorders	57 (33 %)
F5 eating disorders	15 (9 %)
F6 personality disorders	32 (18 %)
5. Additional somatic diagnosis	
None	133 (77 %)
Single	29 (16.5 %)
Double	11 (6 %)
Triple	1 (0.5 %)
6. Number of hospitalizations	
F2 psychotic disorders	37 (54 %)
F3 affective disorders	6 (9 %)
F4 anxiety disorders	4 (6 %)
F5 eating disorders	7 (10 %)
F6 personality disorders	14 (21 %)
7. Therapy	
Pharmacotherapy	116 (67 %)
Psychotherapy	8 (5 %)
Both	25 (14 %)
None	25 (14 %)

### Instruments

The extended version of the Brief Psychiatric Rating Scale (BPRS) was used to assess psychiatric symptoms (Ventura et al. 1993). In the study, the four factor model suggested by Ventura et al. (2000) was applied. The model groups all BPRS symptoms into four subscales: 1. depression (with the items: anxiety, depression, suicidality, guilt), 2. manic symptoms (motor hyperactivity, elevated mood, excitement, distractibility, grandiosity), 3. negative symptoms (blunted affect, motor retardation, emotional withdrawal, self-neglect) and 4. positive symptoms (bizarre behaviour, unusual thought content, disorientation, hallucinations, suspiciousness).

Needs were assessed using the Camberwell Assessment of Needs Short Appraisal Schedule—CANSAS (Slade et al. 1999**)**, a modified version of the Camberwell Assessment of Needs—CAN (Phelan et al. 1995) covering 22 health and social needs. The model suggested by Ruggeri et al. (2004) was implemented, dividing all needs into five domains: basic needs, social needs, functioning, health needs, mental health and social services. For specific needs in each domain please refer to Table 2. The possible ratings for each need were: unmet need (current serious problem, regardless of any help received), met need (no/moderate problem because of help given), no need or not known. Although data were collected from both patients and staff, only the patient-rated data from version CANSAS-P are presented as patient-rated needs are generally regarded in the literature as more reliable. The total unmet needs score is the number of domains with unmet need (0–22).

**Table 2:** CANSAS - needs in domains

Needs domain	Specific needs
1. Basic needs	Accommodation
	Food
2. Social needs	Company
	Intimate relationships
	Sexual expression
3. Functioning	Looking after the home
	Self-care
	Daytime activities
	Childcare
	Basic education
4. Health needs	Physical health
	Psychotic symptoms
	Psychological distress
	Safety to self
	Safety to others
	Alcohol
	Drugs
5. Mental health and social services	Information on condition and treatment
	Telephone
	Transport
	Money
	Benefits

Social functioning was assessed using the second version of the Groningen Social Disability Schedule (GSDS-II) in the form of a semi-structured interview considering local norms (Wiersma et al. 1990). The tool assesses functioning in eight social roles: work, community integration, social relationships, relationship with family, partner and children, household activities and self-care. Functional disabilities were rated on a 4-point scale, where lower scores indicated lesser disability.

### Procedures

Patients meeting including criteria were assessed in outpatient clinics. All researchers were specialists in psychiatry who had received training in verifying diagnoses according to ICD, as well as internal training on the instruments used. The advantage of the study was that the same researcher used all the tools, including BPRS, which excludes researcher bias in the assessment. All patients provided written informed consent and the study was approved by the relevant National Ethics Committee.

### Statistical Analysis

Statistical analyses were conducted in R package 2.4.1 (Becker et al. 1988) using univariable logistic regression models. Dependent variables were numbers of met, unmet and all needs (rated individually and in the domains described above). Independent variables were socio-demographic (age, gender, marital and employment status) and clinical variables (main diagnosis according to ICD-10, number of psychiatric and somatic diagnoses, the length of contact with mental health services, number of hospitalizations, current treatment, the BPRS total score and scores in subscales, the GSDS total score). Total scale scores and subscale scores were counted as the arithmetic mean. With regard to CANSAS, the total number of met, unmet and all needs (as the sum) as well as met needs/unmet needs ratio were also analysed. For correlations of pairs of variables χ^2^ Pearson’s test, Spearman’s rank correlation coefficient and ANOVA tests were used. Hierarchical block regression model was applied to test how blocks of variables influence met, unmet and all needs.

## Results

### Psychopathology

Regarding psychopathology, the total mean BPRS result was 1.6 (SD = 0.4) and there was a significant difference between the total BPRS results in different diagnostic groups. The most severe symptoms were apparent in patients with psychotic (F2) and affective disorders (F3). In terms of the results in BPRS subscales, the most common amongst all patients were depressive and anxiety symptoms (mean result 2.5). Service users with psychotic disorder had the highest scores in subscales of negative, positive and manic symptoms (means respectively 2.2, 1.8 and 1.4). BPRS total results and results in subscales with regard to diagnostic groups are shown in Table 3.

**Table 3:** BPRS total results and results in subscales—mean (SD)

Diagnosis ICD-10	BPRS total result	Manic subscale	Negative subscale	Positive subscale	Depression subscale
F2	1.74 (0.46)	1.38 (0.52)	2.22 (0.88)	1.80 (0.76)	1.83 (0.78)
F3	1.66 (0.42)	1.15 (0.27)	1.55 (0.74)	1.24 (0.50)	3.02 (1.23)
F4	1.44 (0.29)	1.05 (0.16)	1.32 (0.54)	1.08 (0.26)	2.33 (0.79)
F5	1.62 (0.20)	1.04 (0.98)	1.17 (0.20)	1.23 (0.29)	3.17 (0.89)
F6	1.55 (0.29)	1.12 (0.27)	1.27 (0.37)	1.12 (0.25)	2.69 (0.96)
Total	1.58 (0.37)	1.14 (0.31)	1.50 (0.70)	1.26 (0.51)	2.54 (1.04)

### Needs of Patients

The mean total number of needs was 8.1 with met needs 5.0 and unmet needs 3.1. Unmet needs of persons with psychotic, eating, personality and affective disorders (respectively, F2, 3, 5, 6 according to ICD-10) were significantly higher (*p* < 0.01) than the needs of patients with anxiety disorders (F4). Table 4 shows total scores of CANSAS-P with regard to diagnostic groups.

**Table 4:** CANSAS-P total scores with regard to diagnostic groups

Diagnosis ICD-10	Met needs	Unmet needs	Total needs
F2	5.62	3.86	9.48
F3	5.85	3.09	8.95
F4	4.59	2.35	6.94
F5	3.73	3.66	7.40
F6	4.68	3.68	8.37
Total	5.00	3.14	8.14

Regarding needs in five domains, the highest numbers of met and total needs were reported in the health domain. The social needs domain was the most often unmet (1.0) and within this domain the most often unmet need was sexual expression, followed by company and intimate relationships. Furthermore, the mean number of unmet needs in the mental health and social services domain was significantly higher in persons with psychotic disorder than in others. On the other hand, patients with eating, personality and mood disorders more often rated their needs in the health domain as unmet (results respectively 1.1, 1.1 and 1.0). Basic needs were the most often unmet for persons with psychotic and mood disorders (respectively 1.4 and 2.0). For detailed CANSAS-P results in needs domains please refer to Table 5.

**Table 5:** CANSAS-P unmet needs results in domains with regard to diagnostic groups

Diagnosis ICD-10	Basic	Social	Functioning	Health	Services
F2	0.13	1.20	0.75	0.55	0.79
F3	0.19	0.97	0.68	0.95	0.17
F4	0.07	0.79	0.56	0.66	0.21
F5	0	1.26	0.93	1.13	0.26
F6	0.03	1.28	0.75	1.09	0.34
Total	0.09 (0.29)	1.03	0.69	0.83	0.32

### Social Functioning

The mean result of the Groningen Social Disability Schedule (GSDS-II) was 1, which indicates a mild degree of social disability. Results between different diagnostic groups were statistically significant (*p* < 0.001). The highest level of social disability presented in service users diagnosed with psychotic disorders (mean GSDS result 1.4), those with eating and personality disorders functioned at a level similar to patients suffering from affective disorders (1.1), while a diagnosis of neurotic and anxiety disorder was the least socially disabling (0.7). In general, patients’ social functioning was better outside than within the family.

### Variables Associated with Needs

Being single was related to a higher number of total unmet needs (*p* = 0.006) and with regard to the five needs domains, it was strongly correlated with social and functioning needs (results *p* = 0.000). Higher numbers of total met needs as well as total needs and met needs in the services domain were associated with being unemployed.

With regard to main diagnosis, persons with neurotic and anxiety disorder had significantly fewer total unmet needs than others (*p* = 0.006). Unmet needs in the services domain were the highest amongst services users with psychotic disorders (*p* = 0.001), health needs—amongst those diagnosed with affective, eating and personality disorders (*p* = 0.007), while basic needs—amongst patients with affective disorder (*p* = 0.034). The longer patients had been in contact with mental health services, the higher was their number of unmet basic needs (*p* = 0.042). Furthermore, the more hospital admissions patients had had, the more unmet needs they reported in total and in all needs domains.

Total BPRS results and results in subscales were significantly associated with all CANSAS-P total needs results and with the majority of CANSAS-P results in the needs domains. The higher the BPRS results (the more severe psychopatological symptoms), the more needs were reported by patients.

Regarding social functioning, more severe social disability was associated with a higher number of total needs results as well as with unmet needs in the basic, social, health and services domains.

Block regression analyses were carried out to identify the group of variables which would predict the needs of persons with mental disorders. From all variables included in the study, being single, having high positive and depression/anxiety symptomatology, psychiatric hospitalizations and high social disability accounted for 50 % of the variance in level of unmet need.

## Discussion

### Main Findings and Their Comparison with Literature

The study investigated characteristics of outpatients’ needs and their associations with different socio-demographic and clinical variables, including social functioning. The results show more needs reported by Polish service users than those from Italy (Lasalvia et al. 2000; Ruggeri et al. 2004) and Scandinavia (Middleboe et al. 2001) but have a similar ratio of met to unmet needs. Total needs in a British study (Macpherson et al. 2003) were rated higher, however had a smaller percentage of unmet needs. The described discrepancies in needs results could be explained by differences in patients’ diagnoses. Samples from Italian studies (Ruggeri et al. 2004; Lasalvia et al. 2000) have not included persons with eating disorders and had a smaller percentage of patients with personality disorders (respectively 5 and 7 %) in comparison with the Polish sample (18 %). Furthermore, the results could also reflect the current situation in Polish mental health services. Although a gradual increase in mental health and social services is observed, their availability is still insufficient and a significant amount of care is delivered by families and relatives.

With regard to results in the needs domains, similar scores were obtained to Macpherson et al. (2003), indicating the social needs domain (sexual expression, company and intimate relationships) as the most often unmet (1.0). In this study, 70 % of persons reported unmet needs in the social domain (the most often among service users with personality and eating disorders, followed by persons diagnosed with psychotic disorders). These findings partially support the Italian results (Ruggeri and Tansella 2002), in which social needs were more difficult to meet than clinical ones.

Regarding block regression models, the group of variables explaining 50 % variation of total and unmet need was identified. Middleboe et al. (2001) showed that, in their regression model, gender, age, BPRS and GAF results explained only 20 % of variation of unmet needs and 30 % variation of total number of needs.

### Strengths and Limitations

The main strength of the study is the inclusion of patients with a broad spectrum of diagnoses, from F2 to F6 according to ICD-10. Clinical diagnosis was not verified by assessment of a research diagnosis, since community mental health services in general rely on clinical diagnosis. Although the total number of participants in the study was 174, sample size within diagnostic groups still provided sufficient power for the statistical analysis. The majority of service users in the study were women, but there were no significant differences between genders in terms of sociodemographical and clinical variables (apart from the prevalence of eating disorder diagnosis) as well as needs scores; therefore this did not affect the results.

All tools used in the study had had previously established adequate psychometric properties. Needs were assessed from the patients’ perspective, which of course can be very subjective, but evidence available in literature suggests that service users’ rated needs are more clinically relevant.

### Conclusions/Clinical Implications

Our results indicate that unmet needs in the social domain can pose serious problems, not only for persons with psychotic disorders, but also for those diagnosed with personality and eating disorders. These difficulties can have an impact on the ability to form friendships and personal relationships, which may partially explain the higher percentage of single persons amongst persons with personality and eating disorders. In general, the most common unmet need in the social domain was sexual expression, which suggests that mental health service users may often face problems of a sexual nature which are either difficult to tackle or awkward and too intimate to discuss openly with professionals. Therefore, special consideration in assessing and meeting sexual needs in clinical practice may be useful.

One of the interesting findings of the study was that service users with eating and personality disorders had a similar level of total unmet needs to persons diagnosed with psychotic and affective disorders. This may indicate the necessity to improve Polish mental health services in order to provide more adequate care, including therapeutic programs for persons with eating and personality disorders. However, it is worth mentioning that unmet needs may not only indicate weaknesses in health and social care but also reflect self-perceived deficits regardless of the type of disorder. Nonetheless, the best correlates of unmet needs in general were high depression/anxiety symptomatology and social disability. It suggests that in clinical practice the severity of psychopathological symptoms and psychosocial functioning seems to be more useful in identifying service users’ needs than diagnosis. These findings may suggest that current classifications of mental disorders are still imperfect and in need of modification.

In conclusion, the concept of mental health service users’ needs is very complex and correlations of needs with different variables require further research, including also persons suffering from personality and eating disorders. Profound knowledge of patients’ unmet needs could enable providers to focus on areas in which the consumers require special care, which in consequence could have a positive impact on the trajectory of service users’ mental disorders and lead to better outcomes.
